# Supplementary material for: Comparative genomics provides new insights into the diversity, physiology, and sexuality of the only industrially exploited tremellomycete: Phaffia rhodozyma
Source: BMC Genomics. 2016 Nov 9;17:901. doi: 10.1186/s12864-016-3244-7 (PMC5103461; doi:10.1186/s12864-016-3244-7)
Supplement: Additional file 6: — List of orphan genes with links to PFAM (related to Additional file 1: Table S1). (ZIP 1428 kb) [file 12864_2016_3244_MOESM6_ESM.zip › PFAM_UNIQUE/G05213_P.html]

#                                                                            --- full sequence --- -------------- this domain -------------   hmm coord   ali coord   env coord
  
# target name        accession   tlen query name           accession   qlen   E-value  score  bias   #  of  c-Evalue  i-Evalue  score  bias  from    to  from    to  from    to  acc description of target
  
#------------------- ---------- ----- -------------------- ---------- ----- --------- ------ ----- --- --- --------- --------- ------ ----- ----- ----- ----- ----- ----- ----- ---- ---------------------
  
G05213\_P             -            454 CAF-1\_p150           PF11600.3    216       3.4    5.7  10.7   1   1     0.051       6.2    4.8  10.7    17    94    56   135    47   156 0.67 - Chromatin assembly factor 1 complex p150 subunit, N-terminal  
G05213\_P             -            454 DUF4603              PF15376.1   1286         1    5.1   5.5   1   1    0.0007       1.4    4.7   5.5   178   261    51   138    41   146 0.68 - Domain of unknown function (DUF4603)  
G05213\_P             -            454 PAP\_RNA-bind         PF04926.10   157     0.042   12.0   0.7   1   2      0.11   2.1e+02    0.0   0.2    50   117   106   170    95   181 0.56 - Poly(A) polymerase predicted RNA binding domain  
G05213\_P             -            454 PAP\_RNA-bind         PF04926.10   157     0.042   12.0   0.7   2   2   9.3e-05      0.19    9.9   0.0    20    38   315   333   308   369 0.90 - Poly(A) polymerase predicted RNA binding domain  
G05213\_P             -            454 SOBP                 PF15279.1    306      0.67    8.9  10.8   1   2     0.001      0.14   11.2   6.6    83   213    60   196    41   279 0.54 - Sine oculis-binding protein  
G05213\_P             -            454 SOBP                 PF15279.1    306      0.67    8.9  10.8   2   2         4   5.4e+02   -0.6   0.1   216   247   370   402   300   428 0.58 - Sine oculis-binding protein
